# Research participants as ‘pioneers’? Exploring how neurotechnology research is adapting the rhetoric of scientific risk-taking, exploration and trailblazing

**DOI:** 10.1080/09505431.2026.2673139

**Published:** 2026-05-27

**Authors:** Andrew Ivan Brown, Sara Goering, Eran Klein

**Affiliations:** a Centre for Biomedicine, Self and Society, University of Edinburgh, Scotland, UK; b Department of Philosophy, University of Washington, Seattle, WA, USA; c Department of Neurology, Oregon Health & Science University, Portland, OR, USA

**Keywords:** Neuroethics, science and technology studies, qualitative, naming, technology journalism, Neuralink

## Abstract

The theme of pioneers charting new frontiers has long been a staple of scientific and technological discourse in the United States. Scientists, engineers and entrepreneurs perceived as doing groundbreaking work are lauded as pioneers in their fields. In recent years, the application of the pioneer label has expanded to include research participants in clinical trials for neurotechnologies such as Neuralink or other brain-computer interface (BCI) devices: ‘BCI pioneers.’ What might explain this new popular usage of the pioneer label? American science policy in the mid twentieth century drew on the mythos of the American frontier as a way of mobilizing public interest in scientific and technological innovation on a mass scale. One such way of mobilizing interest was to use frontier rhetoric to attribute novelty to scientific undertakings and technological developments: dubbed the new frontiers. A less explored aspect of this history is the application of the pioneer label to patients and research participants in scientific, mostly biomedical, studies. Through the use of frontier rhetoric, articulations of novelty can be attributed to patients and participants, and their participatory practices, including being the first to undergo a new medical procedure, as well as being explorers and trailblazers in their own right. Such rhetoric may be understood as mobilizing support for research participants by acknowledging their bravery, altruism and contributions to science, with implications for participatory science and scientific and technological innovation.

## Introduction

Public interest in implantable neurotechnology saw a significant increase in 2024 after Elon Musk’s neurotechnology start-up, Neuralink, implanted its brain-computer interface (BCI^1^) device into the brain of their first research participant. On January 29th, 2024, Musk wrote, ‘The first human received an implant from Neuralink yesterday and is recovering well. Initial results show promising neuron spike detection.’ Two months later, Neuralink identified this participant as Noland Arbaugh, and posted a video of him playing chess on the computer with his ‘thoughts alone’ (Neuralink, 2024a). The video, which at the time of writing has 96.8 million views, shows Arbaugh controlling the mouse cursor on the screen while his hands sit idly on his legs. His only interaction with the computer is through the BCI device implanted in his brain. In his 90-minute interview with Joe Rogan, Arbaugh claims that the speed and response time of the neural controller ‘blew his mind.’ He says, ‘when I’m thinking it to move in a certain place, sometimes [the neural device is so fast] that [the cursor is] moving before I think it to move’ (quoted in Rogan, 2024). Neuralink also posted a video of Arbaugh playing the Nintendo racing game, Mario Kart, using a combination of his brain device and an assistive controller in his hand (Neuralink, 2024b).

**Figure 1.**
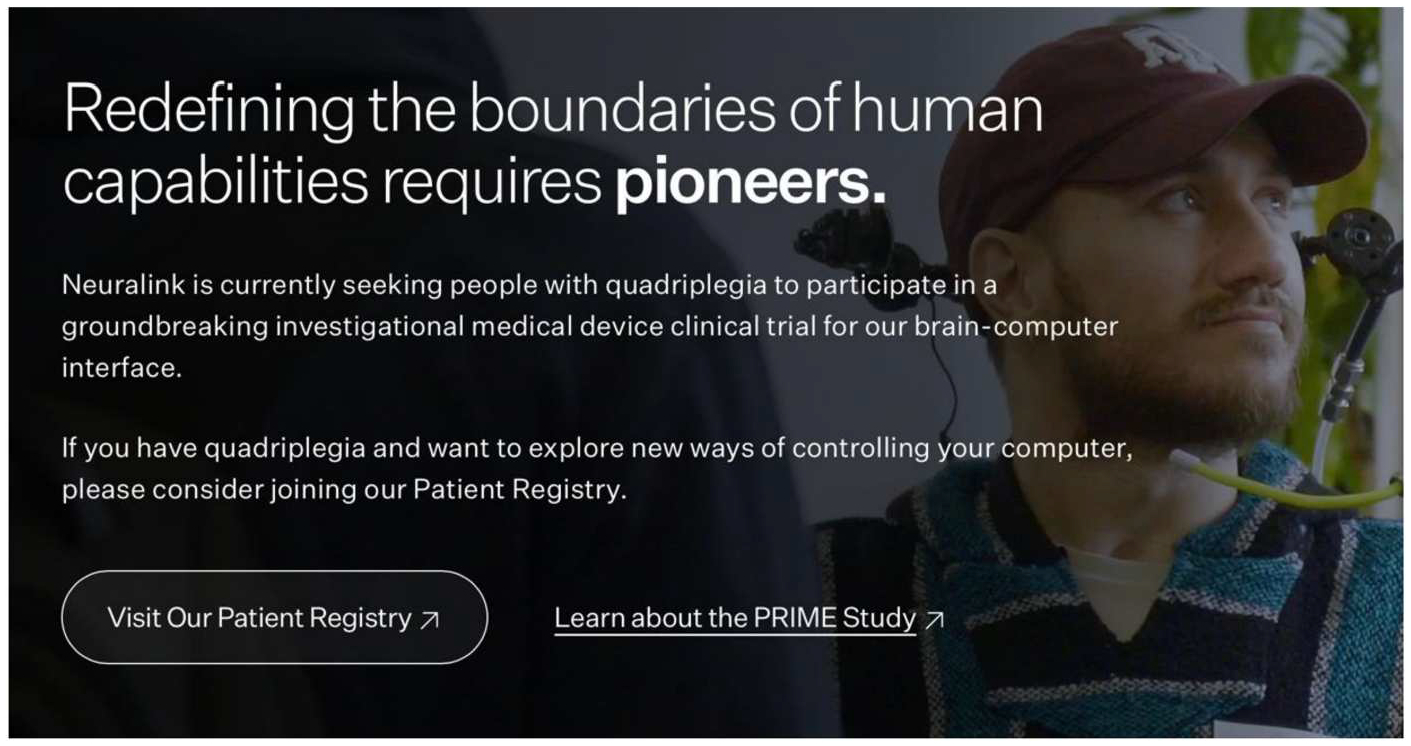
BCI research participant Noland Arbaugh featured on Neuralink’s website’s front page. Source: https://neuralink.com.

**Figure 2.**
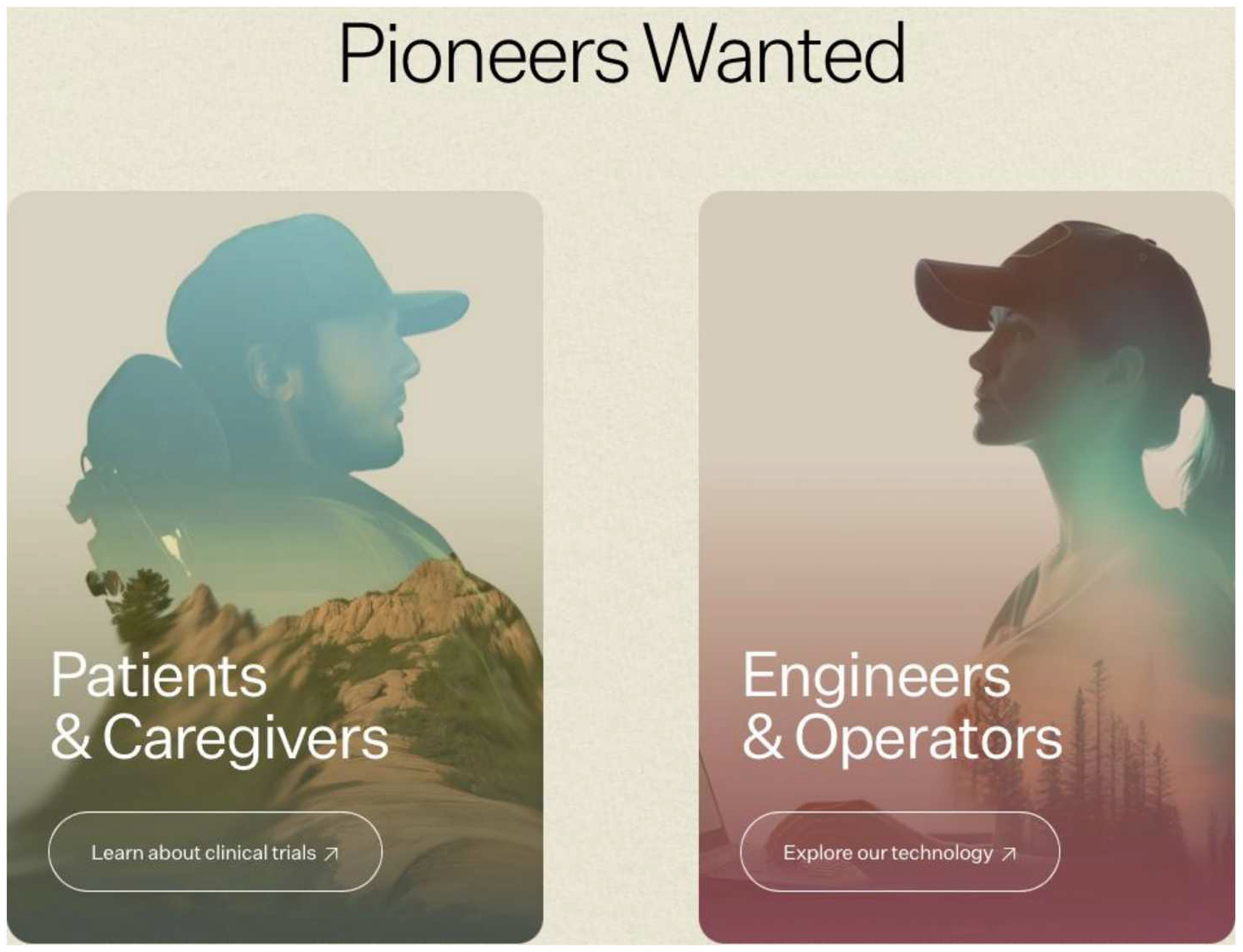
Neuralink’s website’s front page as seen on October 2025 – The left depicts a Mars-like landscape. The right imagery alludes to the American frontier. Source: https://neuralink.com.

Arbaugh became an ‘overnight celebrity’ (Sheriff, 2024, n.p.), reportedly being interviewed over 24 times in the three months following his public reveal (Arbaugh in Sheriff, 2024). In his Rogan interview, Arbaugh says, ‘People keep saying a lot of weird things about me. Like, you know, ‘you’re like an Apollo astronaut.’ I don’t see myself that way. I know people keep saying, ‘you’re the first, you’re like a pioneer.’ I don’t see myself that way at all’ (quoted in Rogan, 2024, n.p.). Nevertheless, the frontpage of Neuralink’s website displayed a photo of Arbaugh overlaid by text which reads, ‘Redefining the boundaries of human capabilities requires *pioneers*’ (the word ‘pioneers’ is bolded) (see Figure 1). In 2025, the website was updated with the simple recruitment phrase, ‘pioneers wanted,’ alluding to patients but also caregivers, engineers and operators involved in the research (see Figure 2).

The motif of pioneers charting new frontiers has long been a staple of scientific and technological discourse in the United States (Spiller, 2015, p. 4). Scientists, medical doctors, engineers and entrepreneurs – such as Musk – perceived as doing groundbreaking research are often lauded as pioneers. But is it weird, as Arbaugh suggests, to label *research participants* as pioneers? Searching online for ‘pioneers in [any given field]’ almost always returns a list of online news articles and blog posts commemorating key researchers, often historical figures, who either ‘pioneered’ the given field or contributed a significant breakthrough to it. Recent examples include the development of mRNA vaccine technology and CRISPR genomic editing. In each of these cases, scientists who broke new ground in these fields were hailed as ‘pioneers’ of the respective technology and went on to receive Nobel Prizes in Physiology for their work (Ledford and Callaway, 2020; Gristwood, 2023). In contrast, it is much more difficult to find instances of patients or research participants being called ‘pioneers.’

Even in well-documented cases of patients’ lay expertise playing essential roles in research, such as the work of HIV patient groups during the AIDS crisis of the 1980s and early 1990s (Collins and Pinch, 2002), the pioneer label tends to be used only to describe the lead researchers, not the wider research staff, and certainly not the patients, research participants or their caregivers (c.f., Bayer and Oppenheimer, 2006). As we will describe in depth below, we did extensive archival newspaper searches to get a sense of how commonly the pioneer label has historically been applied to patients or research participants in public discourse, and we found it to be exceedingly rare.

Participants in neurotechnology research seem to be emerging as a notable exception. They are frequently referred to as ‘bionic pioneers,’ or in the specific case of BCI studies, ‘BCI pioneers.’ For instance, in 2005, long before Neuralink, *Wired* magazine described Matt Nagle, the first participant in Brain-Gate’s ongoing BCI studies (BrainGate, 2024), as a ‘pioneer in the new science of brain implants’; similar to media depictions of Arbaugh, *Wired* made much of Nagle’s ability to play video games (Pong) ‘with his thoughts alone’ (Martin, 2005, n.p.). More recently, another start-up, Blackrock Neurotech, has labeled research participants of its technology^2^ ‘BCI pioneers’ (BN, 2022, n.p.). Some of these participants have formed their own ‘BCI Pioneers Coalition’ (BCIP, 2022, n.p.). What might account for this unusual – maybe liberal – usage of the term ‘pioneer’? Why are neurotechnology research participants being called ‘pioneers’? Is it because using the ‘pioneer’ label is a good way to attract potential participants, ones who seek novelty and are willing to take some risks? Or is it a recognition of the work done by the participants, and the ways they actively contribute to the development of this novel technology?

Claiming to ‘pioneer’ something, or to be a ‘pioneer,’ strongly implies that one is doing something new. Neuralink’s website, for example, describes the company as ‘pioneering brain computer interfaces,’ and Arbaugh brags about how Neuralink’s speed far outpaces competitor BCIs (in Rogan, 2024). Social scientists have argued that ascribing novelty to emerging technologies – whether warranted or not – plays an important role in mobilizing interest in them (Pickersgill, 2011; Galasso *et al*., 2024). Claiming that a technology is doing something new brings in media coverage, builds excitement, attracts funders, and more. Through this sociological lens, it could be argued that Neuralink is using pioneer language to help bolster its claims to novelty, partly as a recruitment strategy. While we do not deny that Neuralink’s use of pioneer language is alluring, we aim to complicate this scholarship – what has come to be known as the ‘sociology of novelty’ (Pickersgill, 2019) – by hypothesizing that *participatory* science is another locus through and for which articulations of novelty are attributed.

In other words, we consider how pioneer language – or more broadly, frontier rhetoric – can help to mobilize support for the participants themselves, in the sense of recognizing their invaluable contributions and promoting them to take on more active participatory roles in scientific and technological innovation. To do this, we take a broader look at the history of frontier rhetoric in media narratives of scientific and technological development throughout the twentieth century, and in BCI research beyond Neuralink, including both the public representations and lived experiences of BCI pioneers. We focus on the understudied cases of patients and research participants being called ‘pioneers’ in these broader contexts. In synthesizing the sociology of novelty with studies in rhetoric, specifically frontier rhetoric, the article intersects with wider debates within medical sociology and science and technology studies (STS) on the role of the promissory – in this case, the participatory promise of working on the frontier – in technological development and participatory science (Brown and Michael, 2003; Martin *et al*., 2008b; Pickersgill, 2011; Tutton, 2011; Broer and Pickersgill, 2015; Brown *et al*., 2016; Galasso *et al*., 2024).

## Analytical perspectives

### American frontier rhetoric as a sociotechnical imaginary

In neuroethics research, the use of the pioneer label is often understood to be fitting if the individual is among the first to use an emerging neurotechnology (Kögel and Wolbring, 2020; Levy *et al*., 2024). While this makes some sense, it does not account for the sociopolitical, historical and cultural contexts that structure how language and rhetoric is deployed in neuroscientific research settings. For example, in the United States, to be a pioneer typically means more than just being the first to do something. The myth of the American frontier (Slotkin, 2000), loaded with ‘toxic ethnocentrism’ (Ceccarelli, 2013, p. 4), portrays backwoods explorers as having charted dangerous, uncivilized territories, taming nature so that future generations could prosper. Pioneers are immortalized in popular fiction like James Cooper’s *Leatherstocking Tales*, and in stories of American folk heroes like Davy Crockett (Spiller, 2015, p. 98). Backwoods frontiersmen are one example of pioneers in American mythology, but there are others. For instance, as John Peck wrote, the ‘real pioneers [were the] men of capital and enterprise,’ the visionary Americans who industrialized the land (quoted in Turner, 1893, para. 12). These entrepreneurial pioneers are seen as taking on financial and reputational risk to carry out their grand visions of industrial expansion. In the field of medicine, British – and later, American – field surgeons working on the frontiers were often called pioneers for embodying the ‘values of Victorian masculinity and heroism’ (Lawrence and Brown, 2016, p. 152).

In her exploration of American frontier rhetoric, Leah Ceccarelli (2013) identifies several characteristics commonly used to describe American pioneers: stalwart and rugged (p. 35); brave, fearless, courageous and bold (pp. 40–44, 49); fiercely individualistic (pp. 35, 41, 44); independent (not bound up with past knowledge or practices) (pp. 38, 40); unconventional (p. 49); willing to make sacrifices (p. 42) and take risks (p. 48); buoyant and exuberant (p. 35); practically minded (p. 35); curious and inventive (p. 36); resourceful (p. 44); and industrious (in the sense of being dedicated and persevering) (p. 46). These characteristics have helped to define what it means to be a pioneer in the United States.

### Modern

American frontier rhetoric developed in the mid-twentieth century, and might best be described as what STS scholars call a ‘sociotechnical imaginary,’ a collective, imagined vision – loaded with cultural idioms, metaphors, language and imagery – of a nation’s past, present and future scientific and technological endeavors (Jasanoff and Kim, 2009, p. 120). Although the concept of the sociotechnical imaginary has been used in inconsistent ways in STS scholarship (McNeil *et al*., 2017), its original usage was meant as a heuristic to compare how different nation states enact culturally-specific rhetorical strategies to sell the importance of scientific and technological innovation to the public (Pfotenhauer and Jasanoff, 2017). This is how we deploy the concept here.

As Ceccarelli argues, while European countries historically conceptualized the ‘frontier’ as a boundary between two nation states (British colonies excepted), the United States mythologized their colonial, imperial past, with its violent erasure of Indigenous populations, to conceive of the frontier as an uncharted, uncivilized and lawless land to be conquered and developed, and its nature tamed (2013, p. 32). In the post World War II period, this myth of the frontier became an ‘imaginative resource’ (Jasanoff and Kim, 2009, p. 141) for the American government to exploit, in order to mobilize public support for national funding toward scientific and technological projects throughout the Cold War (Spiller, 2015, p. 4). Scientific ‘territory’ became spatialized as a metaphorical frontier (Ceccarelli, 2013, p. 12). Funding exploration into ‘uncharted territories’ – like space, but also novel cancer treatment or COVID vaccines – in hopes of scientific discovery often carried with it values and practices bound up in the prior understanding of pioneering: fierce individualism (Ceccarelli, 2013, p. 35, 41, 44), entrepreneurship, free market economies (Spiller, 2015, p. 13) and exploitation (Ceccarelli, 2013, p. 35, 38, 47).

It is from this mid-century sociotechnical imaginary that the modern American pioneers emerged. As Ceccarelli puts it:

[The] motivational appeal to link a scientific career with heroic and exciting work resulted in the transfer of an American pioneering spirit, warts and all, to scientists, molding them in the image of fiercely individualistic, authority-averse archetypes of virile white masculinity – coarse, competitive, and isolated from a fearful public.

(2013, p. 30)

This sociotechnical imaginary tended to individualize the production of scientific knowledge, with CEOs, project leads or heads of laboratories often being hailed as ‘pioneers,’ ‘geniuses’ or ‘visionaries’ whenever their research teams produced pathbreaking work. Such rhetoric usually did not extend to the junior scientists or technicians involved in making these breakthroughs, and especially not to their families or other citizens (Spiller, 2015, p. 12).

While others have analyzed at length how American frontier rhetoric has evolved in scientific and technological discourse (Ceccarelli, 2013; Heggie, 2014; Spiller, 2015; Lawrence and Brown, 2016), nobody, as far as we know, has explored the evolution of the pioneer label as it has been applied to patients and research participants. We aim to fill this gap by looking at the history of naming patients and participants in the United States as pioneers both in the media and through the lived experiences of neurotechnology research participants.

### Applying the sociology of novelty to frontier rhetoric

Social scientists have detailed how hype, hope, expectations, promises and visions of the future all play important roles in mobilizing interest in and support for emerging technologies (Brown, 2003; Borup *et al*., 2006; Martin *et al*., 2008a). This work was later expanded to include novelty as an object of study, given how claims to novelty are often involved in promissory technoscience (Pickersgill, 2021, 2019). The concept of novelty, and what counts as novel, is contested. Accordingly, what makes people perceive a given technology to be ‘new’ is not an intrinsic quality of the technology itself, but a negotiated feature (Pickersgill, 2021). Attributing novelty to an emerging technology – through the use of strategic rhetoric, for instance – can help to differentiate it from relatively similar technologies. Such claims to novelty can modulate expectations of what an emerging technology should do differently and what kind of futures it should help realize. Take Arbaugh for example: he spends much of his interviews detailing what kind of futures BCIs will bring, from allowing paraplegics to drive Teslas (in Rogan, 2024) to helping to colonize Mars (‘well then you’d really have to see yourself as a pioneer,’ the interviewer quips) (in Sheriff, 2024).^3^ In helping to set expectations, articulations of novelty can thus shape the direction and design of emerging technologies; they perform ‘ontological work’ (Pickersgill, 2013, p. 329).

As Martyn Pickersgill describes, the sociology of novelty does not seek to map out what is ‘really’ novel or not, but recognizes novelty as ‘negotiated and attributed through often geographically or institutionally specific forms of sociotechnical praxis’ (2019, p. 617). To our knowledge, however, the sociology of novelty has not yet been applied to American frontier rhetoric. Doing so, we suggest, can provide additional theoretical insights for the field, such as highlighting how novelty can be expressed through a national imagery, and how expressions of novelty can evolve in reaction to major changes in national policy – e.g. Franklin Roosevelt’s mid-century science policy – and events, such as the moon landing.

Much of the promotion around technoscientific novelty occurs in the media (Nelkin, 1996; Brennen *et al*., 2022). For example, in media contexts, Dorothy Nelkin states that ‘to convince their editors about the newsworthiness of a science story, journalists will emphasize the uniqueness of individual events (the “first” discovery, the major “breakthrough”)’ (1996, p. 1601). This kind of exaggerated reporting can transform a ‘micro event’ – one of ‘many little occurrences’ that comprise slow, continuous, technoscientific progress – into an ‘hype event,’ an event that signals a sudden discontinuity or break from ‘normal science’ (Neresini and Lorenzet, 2018, p. 162, 163). Indeed, there is longstanding debate by historians and philosophers on what constitutes and drives scientific progress, including the role of creative or exceptional individuals (Bachelard, 1984; Latour, 2005; Ebke, 2012; Mialet, 2012). Regardless of where one stands in this debate, frequent media narratives of scientific and technological triumphalism may contribute to public perceptions that progress in technoscience happens in leaps and bounds (Neresini and Lorenzet, 2018). Such narratives often reinforce individualistic framings of technoscientific practice by focusing on the lead scientist, often referred to as the ‘pioneer,’ the person who makes the requisite breakthrough for progress to occur.

In applying the sociology of novelty to frontier rhetoric, we thus shed light on how negotiations of novelty happen not only around the technology in question, but the people working on the technology as well. The pioneer label is a key example of how novelty can be attributed to researchers or engineers framed in the media as making ‘breakthroughs,’ but also, importantly, to patients or participants who make invaluable contributions to the research. Taking cues from Pickersgill, we are not interested in questions of whether participants like Arbaugh are ‘really’ pioneers or the research they are involved in are ‘actual’ frontiers. Rather, we are interested in exploring the multiple ways in which novelty is attributed to them and their practices, and the implications of this for participatory science and media narratives of scientific and technological innovation.

In the empirical analysis that follows, we interpret several ways that frontier rhetoric helps to attribute novelty to participants, such as being the first to do something, exploring new territory and ‘trailblazing,’ defined as ‘pointing a new way’, e.g. being a visionary. In our conclusion, we consider how the use of frontier rhetoric grants participants greater recognition for their bravery, altruism and contributions to science – and may help mobilize interest in participatory science, in the sense of encouraging research participants to take on new active roles to assist in the shaping and governance of various scientific and technological endeavors.

## Methodology

Our methodology entails two components: one, archival searches and analysis of media, and two, conducting interviews with participants of implantable neurotechnology studies. We wanted to identify cases where the pioneer label was applied to patients or research participants to get a sense of the history and contemporary usage of this application, including the how, why, and to whom questions. We also wanted to analyze what historical trends or shifts had occurred with this usage. We first tried Googling combinations of the search terms ‘pioneer,’ ‘patient,’ ‘participant’ and ‘recipient’ as well as various novel biomedical technologies or topics that are connected to first-in-human biomedical media coverage, such as ‘pig heart transplant,’ ‘vaccine,’ ‘HIV,’ ‘test tube baby’ and more. This allowed us to pinpoint several popular cases where patients and research participants had been labeled pioneers. This process revealed four umbrella phrases that were commonly used to capture existing cases: ‘pioneer patient,’ ‘implant pioneer,’ ‘bionic pioneer’ and ‘BCI pioneer.’ Only the first of these had any consistent use prior to the 2010s.

Public narratives around a given topic, including how certain terms are used and circulated by the public, are often shaped by the popular press (Fowler, 1991). Thus, to achieve a broader, historical sampling of how the pioneer label has been used, we searched for ‘pioneer patient’ in the *Chronicling America* database, which covers newspapers from each state between 1836–1922, the *Readex America’s Historical Newspapers* collection, covering 1736-present, and the *ProQuest Historical Newspapers* collection, covering the 1880s to 2000s. However, to avoid covering eras where radically different medical methods and paradigms were operative, such as Galenic theory and humorism – which were still well into use until the late nineteenth century (Temkin, 1973) – and to keep our focus on modern scientific theory and practice, we limited our results to only cover cases documented from 1900 onwards, though we recognize that any cutoff will be to some extent arbitrary.

Our findings are presented in Table 1. ‘Year’ represents the first time that the novel descriptor ‘pioneer’ was ascribed to the patient or participant in news media, not necessarily when the novel procedure, study or practice happened. ‘Initial usages’ refers to the number of initial news articles we found that described a patient or participant as a ‘pioneer’ – i.e. was it a one-off mention by a single article or did multiple newspapers use the pioneer label? We defined ‘initial’ as any news article reporting on the same story or event within the first month of the story breaking. The next column, ‘stickiness,’ refers to the number of news articles published at a later date (beyond the first month) that also referred to the patient or participant as a ‘pioneer.’ These are news articles that either provide an update to the story in the months (or often years) following the initial story, or a retrospect years later, yet still used the ‘pioneer’ descriptor, marking the ‘stickiness’ of the term. In both columns, we only counted news articles that explicitly used the ‘pioneer’ language. If an article was written about a particular person, technology or treatment in question but did not use the pioneer label to describe the patient or participant, it was not included in either count.

Textual analysis also included a determination of how ‘pioneer’ was defined or substantiated in these sources, e.g. does it mean ‘first,’ or does it hint at a more complicated use of the term, e.g. involving bravery, risk-taking? We present our results as a representative snapshot of the history, though we recognize that using additional databases and search queries could capture additional cases that we might have missed. We also make no claims to the factual accuracy of the historical cases listed. Our data present how newspaper journalists ascribed novelty to these patients or participants, not whether these procedures were ‘really’ new.

For the interview component, we analyzed publicly available interview data from media interviews with Arbaugh and other participants in BCI research studies, as well as data from our own interviews with BCI participants. As part of a larger study of BCI research participant experience, we interviewed four BCI participants (referred to in this article as BCI-1 through 4), two deep brain stimulation (DBS) participants (for comparison) (DBS-1 and DBS-2) and five support partners (paid caregivers, family members) of participants from both groups (referred to as the ‘SP’ of a given participant, e.g. ‘BCI-1-SP’ for the support partner of BCI-1). For more details on our recruitment and methodology, see Brown *et al*. (2025). For this current project, we asked each interviewee if they considered themselves a ‘pioneer’ (or in the case of support partners, if they considered the participant a ‘pioneer’) and what that term means for them (see Table 2). Interview data we present in this paper also includes their responses to other questions in which they happen to describe themselves or their participation in pioneer-type language.

## Results and discussion

### The history of naming patients and participants in the United States as pioneers

To ‘be the first to do something’ is a common dictionary definition of ‘pioneer,’ especially when used as a verb, and it corresponds to most colloquial usage of the pioneer label. In the United States, however, being called a ‘pioneer’ – the label itself – carries with it additional prestige. Whereas pioneering scientists and medical doctors acquire prestige from being the first to publish a breakthrough finding or develop a new life-saving medical procedure, patients and participants can gain prestige from the pioneer label itself: in the media, they are often praised for their ‘bravery’ (CHOP, 2022, n.p.) and acknowledged as ‘heroes’ (UPI, 2003, n.p.). But there was a time in American history – in the first half of the twentieth century – when the pioneer label, at least in the domain of science and medicine, did not carry with it this additional prestige or heroic imagery (field surgeons being one exception, as noted below). During this period, we found six cases of patients or research participants being called pioneers (see Table 1). Barring one exception from 1935, in none of these cases was the pioneer label used to grant prestige to the patient, or to imply that they had done something heroic or achieved something worthy of praise. It simply meant ‘the first’ (e.g. the first patient in an asylum).

The news article from 1935 was the first evidence we found of frontier rhetoric shifting for patient or participant pioneers.^4^ The article describes Jane Crawford, the first woman to receive an ovariectomy^5^ and survive, as a ‘pioneer patient,’ a ‘pioneer heroine’ and a ‘great pioneer woman,’ the latter phrase referring to her status as a settler (WP, 1935, n.p.). A local doctor is quoted by the news article as likening ‘the “mutual spirit” of the surgeon and the patient with the frontier spirit that won a Western empire’ (WP, 1935, n.p.). This suggests that being the first in a new scientific experiment or medical procedure was now starting to be likened to the feats of American frontiersmen, in this case, field surgeons.

**Figure 3.**
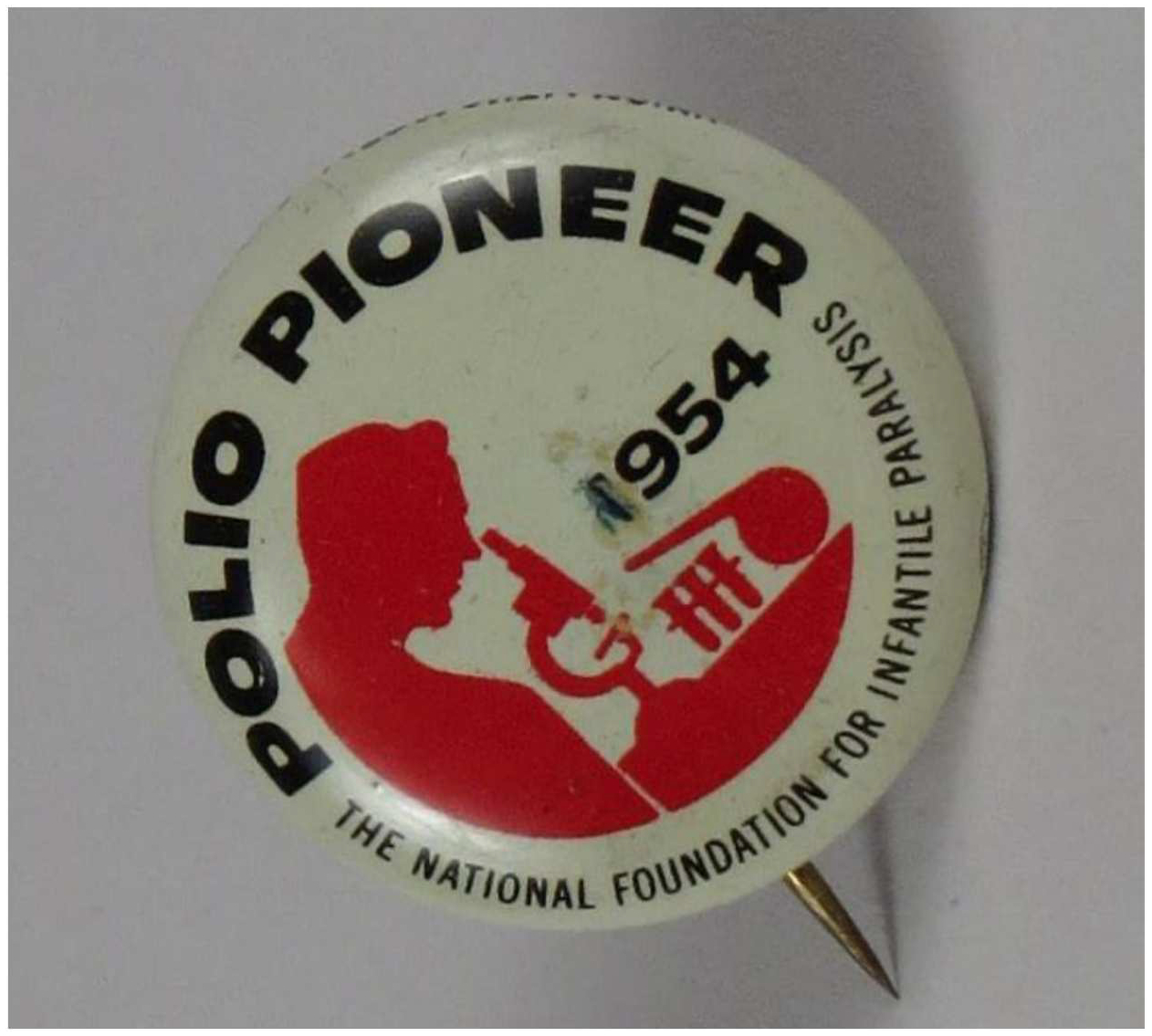
Polio Pioneer Button, given to a participant in the 1954 polio vaccine field trials, object no. NMAH_556623, (National Museum of American History, Smithsonian Institution, Washington, D.C.). Source: Image from Smithsonian Collections online, accessed Feb. 10, 2026, https://americanhistory.si.edu/collections/object/nmah_556623.

This use of frontier rhetoric became cemented as a full-blown national sociotechnical imaginary in 1945 when Vannevar Bush, the director of the Office of Scientific Research and Development for the United States government, published his report titled, *Science – The Endless Frontier*. In it, he stated that ‘the pioneer spirit is still vigorous within this nation [and] Science offers a largely unexplored hinterland for the pioneer who has the tools for his task’ (Spiller, 2015, p. 9). The report, and the rhetoric within, was part of President Franklin Roosevelt’s broader political strategy to gain public support for a progressive, postwar science policy (Ceccarelli, 2013, p. 44). The strategy was widely successful. From then on, to be called a ‘pioneer’ in the United States would almost always carry with it considerable prestige. For example, in 1954, the first children to receive polio vaccines were given buttons with the title of ‘Polio Pioneer’ to attach to their clothes, to show off to others (Lambert and Markel, 2000). In other words, the decision to label these children as pioneers was not simply definitional, as it had been in decades past. Instead, it connected to the larger sociotechnical imaginary of the nation, emphasizing how these children were helping to achieve national goals of scientific and technological progress for the betterment of all humankind (Figure 3).

We see this same pattern repeated in the coming decades. Like the polio pioneers of 1954, the patients or participants in each of the cases we found were granted pioneer status for their bravery, altruism and contributions to science and medicine, garnering them considerable prestige. In 1975, an official government report referred to the first patients of a new diabetes therapy as ‘pioneer patients,’ defining the phrase as patients ‘who, either voluntarily or who through desperation, agree to a new form of treatment or operation to blaze a new path in medical exploration’ (Senate, 1975, p. 30679). This usage of the pioneer label, when applied to patients or participants, remained relatively consistent throughout the second half of the century, in all of the cases we analyzed.

Aside from the polio pioneers, Barney Clark, the first person to receive an artificial heart implant, was the only pioneer patient to receive considerable media attention, with over 2,000 news articles mentioning his status as a pioneer. He underwent the experimental procedure in 1982, and later died from complications in 1983. His is the first case we found where a pioneer’s death played a role in characterizing his status as a pioneer. He was first called a ‘pioneer’ by his medical doctors upon receiving the implant (AC, 1982, n.p.), but this labeling was only mentioned in nine out of approximately 500 news articles that reported on Barney Clark prior to his death. After his death, in contrast, we found over 2,000 news articles that explicitly made reference to his status as a ‘pioneer.’ The media eulogized him as having made a heroic sacrifice for science. This would become a common theme in future cases of patients being called pioneers after their death – usually in their obituaries – including in cases where their death was unrelated to their pioneering work. The 2024 death of implant pioneer Rick Slayman, the first person to receive a genetically-modified pig kidney, is a more recent example of this phenomenon (Afshar *et al*., 2024).

Another change during this period was the cultural imagery of the frontier motif, with space exploration representing the literal image of the new American frontier, and Apollo astronauts representing the literal image of the new American pioneers. This evolving frontier imagery, in a few cases, made its way to pioneering patients and research participants as well. In 1982, paraplegic Nan Davis became the first person to walk using an early prototype of a neuroprosthesis: a computer-controlled device used to stimulate her legs with electricity. Upon walking for the first time as a paraplegic, she stated, ‘I feel like a pioneer. This is history, the first time it’s ever been done’ (Cohn, 1982, p. n.p.), alluding to Neil Armstrong, the first person to walk on the moon. Or to give a more explicit example, the front cover of Jennifer French and James Cavuoto’s 2014 book, *Bionic Pioneers: Brave Neurotech Users Blaze the Trail to New Therapies*, features an illustration of what appears to be Apollo astronauts walking on the moon. Patients and participants in implantable neurotechnology studies also make frequent reference to the moon landing when looking for analogies to their experience (Levy *et al*., 2024, p. 8). For instance, a recent Rolling Stone’s article describes one BCI participant’s experience as follows: ‘It may seem like a small step, but this was a giant leap for Buckwalter, 69, the self-proclaimed “neuronaut”’ (Loucaides, 2025). These are examples of how novelty can be expressed through the use of imagery.

Among the popular^6^ cases we found of patients or participants being called ‘pioneers’ in the last two decades are the ‘bionic pioneers’ – broadly construed as the first patients and participants to use novel neurotechnologies (French and Cavuoto, 2014) – and the ‘BCI pioneers,’ a label specifically used for patients or participants using implantable BCIs. Possibly influenced by the story of ‘bionic pioneer’ Hugh Herr (Schwartz, 2013), whose experience as a disabled person helped him innovate his own bionic prostheses, French explicitly links bionic pioneering to the role of the innovator or visionary, or what we are calling the ‘trailblazer,’ defined as ‘pointing a new way’ (Merriam-Webster, 2024):

Pioneers are not made; they are missioned. Conventional thinking points to scientists, engineers, and entrepreneurs as the sources of innovation. They definitely have a hand in the process, but we often forget the influence that the end-user, or consumer, has on the technology innovation process.

(French, 2020)

**Figure 4.**
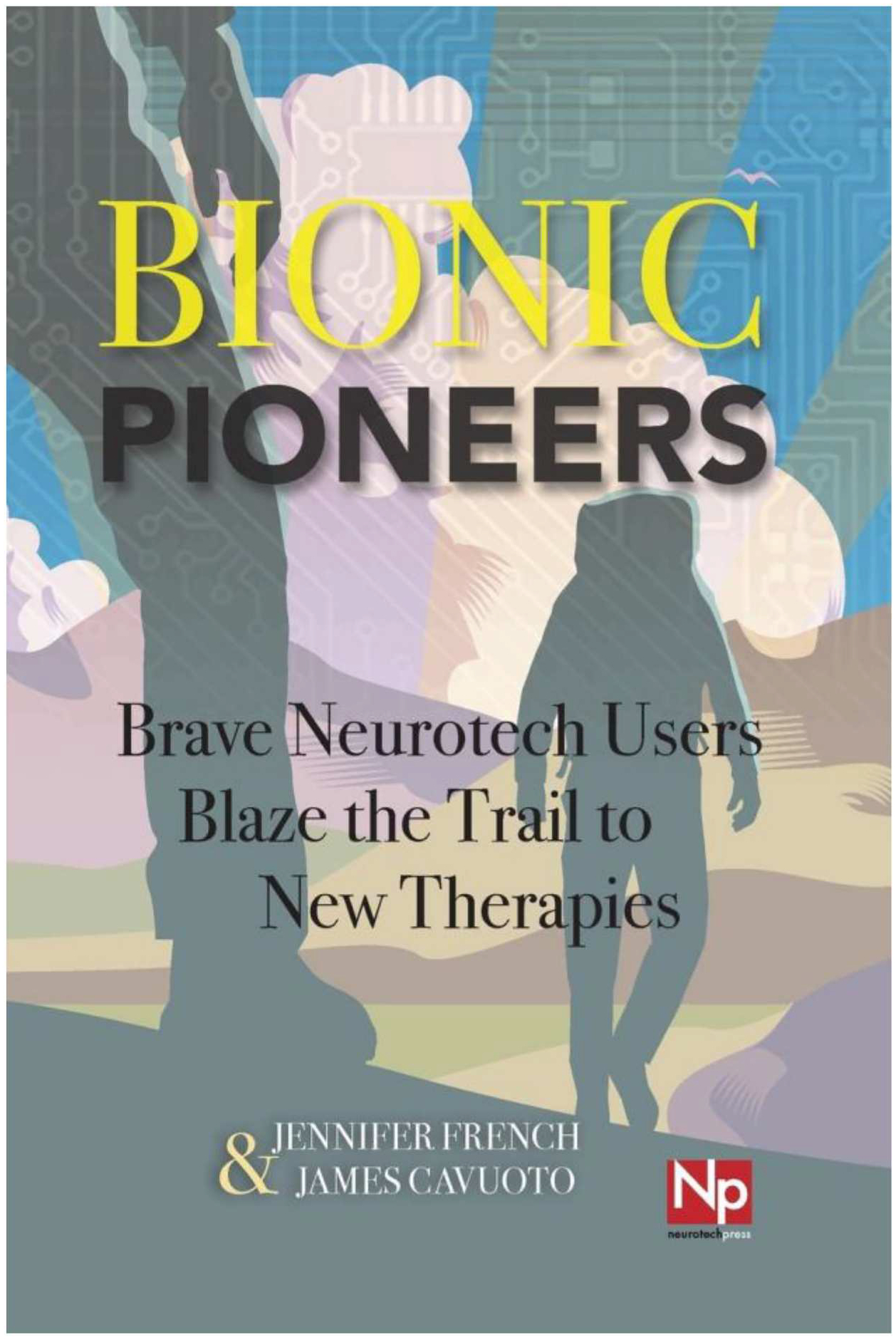
Cover of Jennifer French and James Cavuoto’s 2014 book, Bionic Pioneers: Brave Neurotech Users Blaze the Trail to New Therapies. Neurotech Press. Source: Cover design by Andrew Shalat.

French’s description corresponds to another way that frontier rhetoric articulates novelty, in the sense of offering new visions of the future that technoscience should strive to realize (Figure 4).

As our analysis in the next section will demonstrate, neurotechnology research participants may take on at times multiple or all of the roles that have previously been associated with pioneer patients and participants, including being the first, exploring new territory, risk-taking, altruism and trailblazing, as well as signaling additional connotations like being the first woman in science and experiencing the world in new ways.

### Exploring the use of frontier rhetoric in the localized context of BCI research

In the previous section, our empirics provided a rough sketch of the history of patient and participant pioneering in the United States. We analyzed these empirics to unpack multiple ways that frontier rhetoric attributes novelty to participants and their practices and emphasizes their bravery, altruism and contributions. In this section, we consider what it means to be a pioneer participant in the localized context of BCI research. By analyzing data from interviews we conducted with implantable neurotechnology participants and their caregivers, we offer more fine-grained examples of how frontier rhetoric operates in this localized context. We find that these participants utilize frontier rhetoric to attribute novelty to themselves and their practices (see Table 2 for a summary). We also find that frontier rhetoric may, in some instances, help to reframe pioneering as a collective enterprise, while in other instances, reinforce its individualism.

Implantable neurotechnology research requires participants to undergo brain surgery – an endeavor that, similar to ‘implant pioneers,’ requires participants to take on personal risks for the advancement of science and the betterment of humankind. One participant, upon reflecting on why she might consider herself a pioneer, says, ‘I have always just focused on the ability to persevere. […] I was brave. [For the brain surgery] I had to be willing to be bolted to a table awake all day’ (DBS-1). Similarly, BCI participant Noland Arbaugh claims that he likes ‘to be the one taking all the risks’ so that if something goes wrong, they can fix it for the next person (quoted in Sheriff, 2024).

The symbolic significance of the brain in Western cultures (Vidal and Ortega, 2017) may contribute to neurotechnology research being more conducive to frontier rhetoric than, say, many other kinds of clinical research trials. Most notably, BCI research participants describe the brain as the new frontier: ‘I still believe the great frontier is our brain’ (BCI-4). They use their BCI to explore new ‘territory’ (BCI-2) of the brain. This rhetoric of the brain-as-frontier was used by President Barack Obama when he announced the Brain Research through Advancing Innovation (BRAIN) Initiative (Ferenstein, 2013), and continues to be operationalized in National Institutes of Health (NIH) grant projects and academic articles on BCI research, including our recent Hastings Center’s report that refers to BCI participants as ‘Brain Pioneers’ (Goering *et al*., 2024; see also Kögel and Wolbring, 2020).

Participants also attribute novelty to their practices by describing their use of BCIs as a new material mode of interacting with the world. Consider, for example, how the Apollo spacecraft contributed to a novel experience of pioneering by allowing astronauts to step onto the moon for the first time. Similarly, as one BCI research participant puts it, ‘the mind is a fascinating frontier that I am just diggin’ into. […] I’m just as much as a pioneer as the first man on the moon’ (BCI-3). Participants describe the experience of BCI pioneering as follows: ‘I think that usin’ a BCI, for me, it awakens a second avenue of creative processes’ (BCI-3); ‘using the BCI device is like, it’s a different experience’ (BCI-1); and ‘my experience is unlike anybody else’s in this world’ (BCI-3). According to participants, being able to interact with the world simply through one’s active visual imagery – trained with a BCI device to recognize associated neural activity patterns – offers them novel material affordances.

By utilizing the BCI as a technology of exploration, and the mind as the frontier to be explored with this technology, these pioneers become ‘trailblazers’ (BCI-3). For example, BCI-3 describes how his experience of making artwork using his device enables him to envision new futures for the mind. He invites us to imagine these futures, too:

Think about it. What if you were able to move a brush with your thoughts, how would you feel just drawin’ a couple of lines with your mind? […] The possibility of that could be something in the near future that can happen for you, but it’s already happening for me. […] We’re trailblazing over here. Just by showin’ people that this is possible now, currently, it’s openin’ up the world to be able to dream a little bigger.

(BCI-3)

He sees himself as a trailblazer who uses his BCI to explore new frontiers of the mind, allowing him to construct new visions of future to share with others so that they can ‘dream a little bigger.’ This is something that CEOs, project leads or heads of laboratories cannot do, because only the participants have direct access to this new frontier, though clearly they rely on the expertise of the scientific team to gain that access.

Conventionally, the language and rhetoric surrounding American pioneers suggest a kind of radical individual achievement. However, this conventional, individualistic picture of a pioneer who takes on risks, charts new territories and offers entrepreneurial visions leaves out the teamwork and support structures that encapsulate much BCI research. In the words of Courtney Addison, ‘work from the history of science and feminist STS has, of course, shown that “discovery” is rarely an individual process, and masculinist narratives of scientist-as-hero reinforce an unhelpfully gendered image of science’ (Addison, 2020, p. 152). For example, BCI research trials are notable for their duration, often spanning years, with participants sometimes attending research sessions multiple times each week for several hours a day. During these sessions, they work closely with researchers. Brown *et al*. (2025) describe how BCI research participants are often informally included as members of the research team, and considered ‘coworkers’ by the researchers.

To feel like one is part of the team may contribute to local reframings of frontier rhetoric, where pioneering can be understood as a collective enterprise, e.g. research participants as playing key roles in a pioneering team. For example, BCI-4 makes mention of the ‘covered wagons’ that allowed early American pioneers to cross the country, harkening back to the families and communities that collectively partook in the pioneering work of early settlers (BCI-4). As historian James Spiller puts it, ‘male explorers would be followed by pioneering families’ (2015, p. 83). BCI-4 also describes how she and the researchers associated the neural data being recorded on the computer screen with frontier imagery: ‘You know, when you flick on the computer and those neurons start making noise and jumping around, it was like landing on the moon for us’ (BCI-4). This description bears striking resemblance to the experience of turning on a television set in 1969 to see Apollo astronauts speaking live to the audience and jumping around on the moon, or being a scientist in the NASA control room watching the fruition of their team’s research and hard work finally paying off. Given that researchers doing groundbreaking work on the frontiers of science are often perceived to be pioneers, it makes sense that these participants feel like pioneers too, if they view themselves as part of the research team. The use of pronouns in some of their descriptions is telling in this regard: ‘it was like landing on the moon for *us*’ (BCI-4) and ‘this is *our* frontier, to understand the brain’ (BCI-4).

## Conclusion

We began this article by discussing media coverage of Neuralink’s first participant, Noland Arbaugh, who is presented as a ‘pioneer.’ Despite Arbaugh’s stated discomfort with the label, Neuralink frames him as a pioneer on their website, and invites other pioneers – patients, caregivers, engineers and operators – to consider enrolling in a clinical study or applying for a job with Neuralink. What might account, we asked, for this unusual usage of the term ‘pioneer’? In a basic definitional sense, ‘to pioneer something’ indicates that a person is doing or developing something new. In looking to the sociology of novelty, we considered the possibility that Neuralink is using pioneer language to do novelty work; that is, by claiming to pioneer BCIs, calling Arbaugh a pioneer and deploying frontier imagery on their website, Neuralink might be trying to bolster its claims to novelty as a way of mobilizing support for the company and technology.

However, in exploring the broader history of how the pioneer label has been applied to patients or participants in the United States in twentieth century mass media, and in the lived experiences of BCI participants in studies beyond Neuralink, we have complicated this scholarship. It may be true, especially in the Neuralink example, that labeling research participants as pioneers sets an expectation that future participants will get to do promising work on the frontiers of science and become pioneers themselves. We do not deny the allure of being a pioneer, but our findings suggest that what is encapsulated in that promise is the novelty of being an *active* participant in these research studies. Our previous work, for example, has demonstrated how BCI researchers and participants view each other as ‘coworkers’ (Brown *et al*., 2025). This is a radical transformative view of what it means to participate in a clinical study.

Our suggestion that labelling patients or research participants as ‘pioneers’ signals the novelty of their active participation in scientific and technological development may help to explain the sparsity of such labelling throughout twentieth century mass media, especially compared to how often lead scientists, heads of labs and entrepreneurs are granted the pioneer status. The American brand of frontier rhetoric was largely developed out of the mythologization of the American frontier, and tends to individualize ‘heroic’ pioneers who conquered the land, either through backwoods exploration or industrial expansion. As a sociotechnical imaginary, American frontier rhetoric is one of many historical factors that contributed to the conventional framing of scientific knowledge production as an individual enterprise (see Addison, 2020, p. 152), and likely helps to explain why patients and research participants were scarcely granted the pioneer status. The media has a tendency to hype up developments in technoscience, thereby contributing to narratives of scientific and technological triumphalism. These narratives rely on individualistic framings of technoscientific progress: that breakthroughs typically happen because of an exceptional individual or genius – usually the lead scientist, engineer, or entrepreneur of the company – and often named a ‘pioneer.’

BCI participants, however, are a notable exception. Like their pioneer patient predecessors, they take on personal risks – e.g. undergo brain surgery – so that they can contribute to research that makes no promise of providing clinical benefit. They are also a notably small population, so their individual contributions are all the more valuable to researchers whom have limited data to work with compared to much larger pools of participants in other kinds of clinical studies. This may also mean their contributions garner considerable recognition from researchers and the public. The cultural image of the brain as a new frontier and the compelling articulations of novelty described by participants of their experiences using a BCI (e.g. doing things with thoughts) are also likely enmeshed in what it means to be a BCI pioneer. All of these qualities are admirable in their own right, and when taken together, paint a picture of active participation. BCI researchers, for example, often consider participants to be part of the research team, as do the participants themselves (Brown *et al*., 2025).

This pioneer labeling may thus reflect an emerging landscape of technological governance that uses frontier rhetoric to recognize what regular people, as participants or patients, can add to science, and acknowledging the role of the wider team so that it is not just the lead scientists or entrepreneurs who get all the honor and recognition.

Our findings show that, at least in a few notable cases like the Polio Pioneers, attributing novelty to patients or participants was a way of acknowledging their bravery, altruism or contributions to the science. These are early historical examples of what may increasingly become the norm in coming years – as STS scholars have noted, in recent years there has been a largescale shift by Western governments and corporations to reshape their relationships with citizens by giving them more of a governing role in scientific and technological development (Macq *et al*., 2021, pp. 161–162). The aforementioned BCI Pioneers Coalition may be considered an example of this; their website describes them as a group of BCI participants that works ‘to establish a set of ethics, guidelines and best practices for future patients, clinicians and commercial entities engaging with BCI research studies’ (BCIP, 2022, n.p.). The creation of these new governing roles have been informed by the rise of participatory frameworks in science policy (Doezema and Frahm, 2023), patient engagement in healthcare (Lindén, 2021; Gunn *et al*., 2023; Simoncelli, 2023) and co-creation frameworks in technology (Macq *et al*., 2021; Lipp *et al*., 2023).

Notably, one of the new governing roles may entail citizens becoming visionaries themselves, and engaging their visions with other members of the public (Hilgartner, 2015, p. 34; Knopf *et al*., 2023, p. 3; Lipp *et al*., 2023, p. 153). Just as Americans have long held a fascination with watching astronauts explore new worlds on television, there may be substantial public interest in learning about the journeys and experiences of BCI pioneers as they do their work on the frontiers of the brain. Directing attention to the participants and their pioneering activities may encourage more recognition for their active contributions and their valuable expertise.

Our analysis of twentieth-century media suggests that individualistic framings of pioneering scientists overshadows the rare instances of journalists reporting on patient or participant pioneers. However, with the changing media landscape of the twenty-first century, there may be ample opportunities for fresh usages of frontier rhetoric to take place. For example, Hans Peters argues that, in recent years, science communication is increasingly shifting away from traditional journalists toward ‘citizen journalists,’ such as popular science bloggers and YouTubers (Peters, 2013, p. 14108). These voices can also include scientists who communicate to the public directly through their own social media channels, as well as, we suggest, *research participants* who take on public-facing science communication roles.

Given frontier rhetoric’s unsavory ties to settler colonialism as well as its toxic ethnocentrism, we can understand why some have argued, as Ceccarelli does, that scientists should actively try to stop using this rhetoric (2013, pp. 3, 4). While we appreciate this critique, it is worth considering the benefits that frontier rhetoric could have for mobilizing support for participatory science. This could include the expectation that doing pioneering scientific work will be viewed as a team effort rather than an individual one, and that participants will receive recognition for the work that they do. That said, there may be some slippage regarding how effective the pioneer label can be in shaping more collectivist narratives of technoscientific progress, given its historical and cultural embeddedness in American individualism, and its association with radical individual achievement.

## Figures and Tables

**Table 1.** List of historical cases where patients or participants were called ‘pioneers,’ 1900–2024^a^.

Year^b^	Name of pioneer	First patient or participant of … / First transplant recipient of …	‘Initial usages’ # of initial articles	‘Stickiness’# of future articles (years published)	Reference (for earliest article found)
1900	Unnamed	smallpox in a Dawson hospital	n/a	n/a	(MA, 1900)
1911	Charles Behle	the Washington Asylum	1	0	(WT, 1911)
1917	Harry O’Neill	a new Fargo hospital	1	0	(Harry O’Neill, 1917)
1924	Marion Tyler	a new, plastic surgical operation for ankles	1	0	(BAH, 1924)
1935	Jane Todd Crawford	a ovariectomy	2	7 (1959–2024)	(WP, 1935)
1946	Gertrude von Costenoble	a successful leprosy treatment in Oregon	2	0	(Oregonian, 1946)
1954	‘Polio Pioneers’	a new polio vaccine	27	~2,500 (1956–2021)	(NYT, 1954)
1966	Raymond Wilmer	a deceased person’s heart valve	1	0	(CDDDE, 1966)
1968	George Debord	a heart	1	0	(LAT, 1968)
1974	Verna Shearer (one of several)	a new type of pacemaker	1	0	(SDC, 1974)
1975	Aileen Paskoff	diabetes therapy	1	1 (1988)	(Senate, 1975)
1976	Jackie McCreadie (one of several)	a kidney	1	0	(Barber, 1976)
1982	Nan Davis	a computer-controlled device for electrical leg stimulation	1	0	(Cohn, 1982)
1982	Barney Clark	an artificial (mechanical) heart	9	~2,000 (1983–2021)	(AC, 1982)
1988	Scott Headding (obituary)	a heart at a new transplant center	1	0	(Kiger, 1988)
1990	Cindy Martin (obituary)	a heart, liver and kidney	1	0	(Newsday, 1990)
1990	Tony Mascio	a new treatment for AIDS	1	0	(TP, 1990)
1991	Robert Wright (obituary)	open heart surgery	1	0	(Vincent, 1991)
1992	Esther Kilcup (obituary)	a kidney	1	0	(SPI, 1992)
1993	Unnamed	a gene therapy for cystic fibrosis	n/a	n/a	(Angier, 1993)
1994	Diane Lea (one of several)	Rheumatoid arthritis research	2	0	(Taylor, 1994)
1994	Vivian Wingate	a titanium valve in 1968	1	0	(Lackey, 1994)
1994	Clarence Miller (obituary)	coronary bypass surgery	1	0	(NFDN, 1994)
1995	Unnamed	gene therapy for inborn immune deficiency disease	n/a	n/a	(Cooke, 1995)
1996	Rebecca Lilly	gene therapy for a brain tumor	1	0	(Parcell, 1996)
1999	Babe Ruth	chemotherapy	1	1 (2021)	(Altman, 1999)
**Notable cases in the twenty-first century (for reference**)^c^					
2001	Robert Tools	an artificial heart	16	~60+ (2001–2023)	(SDUT, 2001)
2005	Matt Nagle	an implantable BCI trial	10	~10 (2006–2024)	(Martin, 2005)
2009	‘BCI Pioneers’	implantable BCI trials	1	~600+ (2020–2024)	(Keim, 2009)
2014	‘Bionic Pioneers’	neurotechnology	1	~400 (2014–2024)	(French and Cavuoto, 2014)
2019	Victoria Gray	CRISPR gene-editing treatment	10	~150+ (2020–2024)	(WAMU, 2019)

a It cannot be stressed enough just how common it is in the United States to apply the pioneer label to scientists, medical doctors, engineers, entrepreneurs and other individuals perceived as doing groundbreaking work. So while this list might look long at first glance, that we could almost fit it into a single page indicates just how rarely the label has been applied to participants and patients.

b Year refers to when the first search result showed up for each instance of the pioneer label being applied, not when the study or medical procedure took place. In instances explicitly labeled as obituaries, we found no evidence that the pioneer label had been used prior to the individual’s death.

c Our full results are only listed for the years between 1879–1999. With the increasing use of ‘pioneer patient’ in obituaries, we decided the table would be too long if we continued it into the 2000s. A few notable instances from the 2000s to 2024 were added for reference. Six instances (prior to the year 2000) were excluded due to not being based in the USA.

**Table 2.** Interviewee responses to the question, ‘What does being a pioneer mean for you?’

Pioneering themes	Responses^a^
Rejecting pioneer label	[The pioneer label is] not one I’ve used or had used. I would only expect that to be used in relation to [the lead researcher]. (DBS-1)
Being the first	Well, I’m the only one in – I think there’s other people now, but for one time I was the only one with the cortical leads and the deep leads at the same time, so I literally was a pioneer. (DBS-2) He tells me he was the first. (DBS-2-SP) was the first. (BCI-1)
Risk-taking	I was brave. [For the brain surgery] I had to be willing to be bolted to a table awake all day. (DBS-1)
Exploring new territory	Well, that’s the name they’re sticking on us. […] It’s just someone who is going forward into new territory. (BCI-2) I’m just as much as a pioneer as the first man on the moon. (BCI-3) It made me think of astronauts. They were pioneers. The people that crossed the country in those covered wagons. I think of them as pioneers, so I guess in a sense, we’re the modern-day pioneers. (BCI-4)
Trailblazing (‘pointing a new way’)	We’re trailblazing over here just by showin’ people that this is possible now. It’s openin’ up the world to be able to dream a little bigger. (BCI-3) A trendsetter. (BCI-4-SP) I would say she’s a pioneer in the sense of bein’ able to bring forth the knowledge that most people would not be aware of or not have accessibility to. This is outer space type stuff for most people, and her bringin’ it to an earth level, I think, is what she feels like she’s bringin’ to the party. (DBS-1-SP)
Being a woman in science	Put me down in the pioneer encyclopedia. […] I want my grandchildren, hopefully, one day to know what their grandmother did and why she did it. I did it for the very reason that I believe in the research. I believe in science, and I believe that women need to be represented in the science as well. (BCI-4)

a DBS 1–2 = Deep brain stimulation patients (n = 2); BCI 1–4 = Brain-computer interface participants (n = 4); SP = support partner.

## Footnotes

1. Throughout this article, we use the acronym “BCIs” for implantable BCI devices.

2. Blackrock Neurotech engineers BCI devices but, unlike Neuralink, clinical trials are conducted in partnership with external research institutions using federal grants, such as universities.

3. Many, though not all, of Arbaugh’s visions allude to what we might playfully call the ‘Musk imaginary’ – the CEO’s own imagination distributed and collectivized (Mialet, 2008) across his followers.

4. In her book, *On the Frontier of Science: An American Rhetoric of Exploration and Exploitation*, Leah Ceccarelli claims that frontier rhetoric underwent a significant change sometime between the 1930s and 1940s, before it became what we would consider a full-blown national sociotechnical imaginary starting in 1945. She traces its origins to a book by Frederik Turner in 1893, who in turn was likely influenced by Francis Bacon’s work in the early 1600s (2013, pp. 30, 31).

5. A surgical procedure in which the ovaries are removed. There is a dark history of ovariotomies being used to forcefully sterilize women. This was not the case for Crawford, who needed the surgery to survive.

6. Our use of the qualifier ‘popular’ here should be taken loosely, as traditional newspaper databases are not a good indicator of popular usage of a term in the era of social media.
